# Effects of dietary microbial feed supplement on production efficacy in lactating dairy cows

**DOI:** 10.3168/jdsc.2020-0002

**Published:** 2021-03-12

**Authors:** B.M. Goetz, J. Lefler, M.A. Abeyta, E.A. Horst, E.J. Mayorga, M. Al-Qaisi, S. Rodriguez-Jimenez, C. Martino, A. Izzo, R. La, H.B. Green, C.E. Moore, M. Embree, L.H. Baumgard

**Affiliations:** 1 Department of Animal Science, Iowa State University, Ames 50011; 2 Ascus Biosciences Inc., San Diego, CA 92121

## Abstract

This study evaluated the effects of a microbial feed supplement (MFS; Galaxis, Ascus Biosciences Inc.) comprising 2 native rumen microbes on performance parameters in mid-lactation dairy cows. Forty-six lactating primiparous and multiparous Holstein cows [629 ± 62 kg of body weight, mean ± standard deviation (SD); parity 1.64 ± 0.49; 119 ± 38 days in milk; 45.11 ± 3.81 and 52.73 ± 4.77 kg/d of milk yield for primiparous and multiparous, respectively] were enrolled in a study containing 3 experimental periods (P). During all periods, enrolled cows were fed the same base total mixed ration (TMR) ad libitum once daily. During P1 (7 d), baseline data were obtained for covariate analysis. At the beginning of P2 (60 d), cows were assigned to 1 of 2 dietary treatment groups in a randomized complete block design to balance for milk yield (MY), parity, and days in milk: (1) a control diet (CON; base TMR; n = 23), or (2) a control diet supplemented with 5 g/d of MFS (MFS; n = 23). Sample size was determined based on previous, unpublished results involving this MFS; a 3-kg difference between groups with a SD of 3.5 kg could be detected with sufficient power (0.81) using a total sample size of 46 cows. Treatment was top-dressed and hand-mixed into the top one-third of the TMR. During P3 (7 d), no treatment was administered, and all cows were fed the base TMR. When analyzing all cows in the data set, MFS had little to no effect on performance. However, modeling revealed that the fixed effect of covariate milk production level had a significant effect on the response of MY and ECM, and further investigation of the data revealed that treatment effectiveness in P2 correlated with milk production during P1. Cows were retrospectively categorized into 2 milk production groups (MPG) balanced for parity: MPG1 (i.e., <53 kg/d of ECM during P1; n = 34) or MPG2 (i.e., ≥53 kg/d of ECM during P1; n = 12). Energy-corrected milk was increased by 4.4% in MFS-administered MPG1 cows compared with CON cows during P2. Although there were no significant effects of MFS on production variables for MPG2 cows, MY tended to be decreased by 3.9% in MFS-administered cows compared with CON cows. Further investigation is needed to understand production level response differences and the effect of supplemented native rumen microbes on animal health and productivity.

## Highlights

• Microbial feed supplementation (MFS) had differing effects based on production level.
• MFS increased production in cows with initial production below 53 kg/d.
• MFS tended to alter milk composition differently dependent upon production level.

## Graphical Abstract

**Figure:**
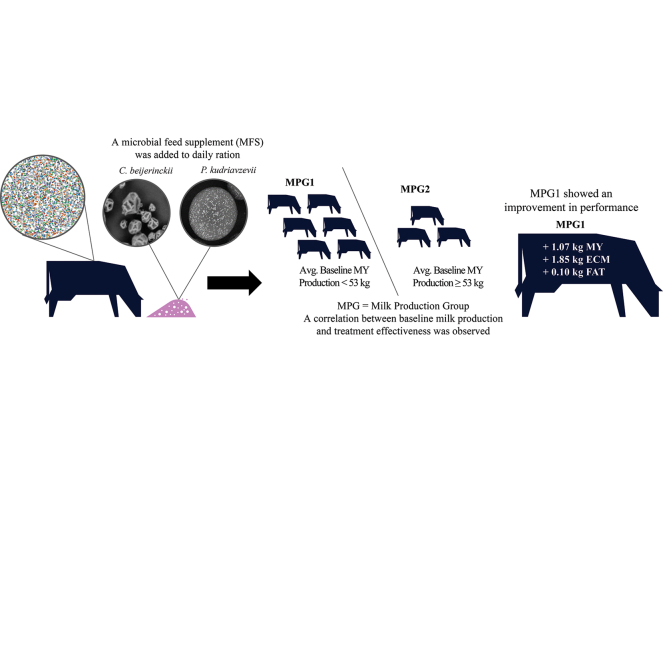
**Summary:** Conventional dairy probiotics often comprise microbes isolated from the environment, not from the gastrointestinal tracts of ruminants; thus, their capacity to influence cow performance is limited. We tested the effects of dietary microbial feed supplementation on production efficiency and quality in lactating dairy cows using microbes native to the rumen environment. Microbial feed supplementation (MFS) had little to no effect when all cows were considered. However, a retrospective analysis demonstrated that lower-producing cows had increased energy-corrected milk and tended to have increased milk and fat yields, along with increased lactose percentage over time, whereas higher-producing cows had decreased milk yield, increased milk protein percentage, and increased body condition score when fed MFS.

Current direct-fed microbials (**DFM**) are livestock feed additives that contain microorganisms in the form of fungi or bacteria (Seo et al., 2010). Numerous studies investigating the influence of DFMs on dairy cow efficiencies have been conducted (Desnoyers et al., 2009; Poppy et al., 2012; Ferraretto and Shaver, 2015). Furthermore, DFM supplementation has been shown to provide potential health benefits to the host, such as reducing the risk of SARA (Ghorbani et al., 2002) or stress-induced diarrhea in calves (Kim et al., 2011). However, there are many discrepancies in the effectiveness and consistency of DFMs on performance and health parameters (Uyeno et al., 2015; Retta, 2016). Direct-fed microbials often comprise microbial strains on the American Association of Feed Control Officials' authorized listing of Direct-Fed Microorganisms (AAFCO, 1999). The bacteria and fungi on this list have a long history of consumption and use in food, such as *Saccharomyces cerevisiae* (bread yeast) and *Lactobacillus acidophilus* (yogurt inoculant). Because many of these strains are not native to the rumen (Kamra, 2005; Kothari et al., 2018), they have limited ability to manipulate and interact with the rumen and its native microbial community (Moraïs and Mizrahi, 2019). In this study, we explored the effectiveness of a microbial feed supplement (**MFS**) comprising 2 live native rumen microorganisms on dairy cow performance. The MFS is composed of *Clostridium beijerinckii* DAIRY20 and *Pichia kudriavzevii* DAIRY21, 2 microorganisms that were originally isolated from the rumens of high-performing Holstein dairy cows consuming a commercially relevant TMR. These strains were selected by analyzing the rumen microbiome across highly productive dairy cows under a variety of feed regimens and locations (Zengler and Embree, 2016). In vitro, the 2 strains have been shown to enhance cellulose digestibility and generation of volatile fatty acids in medium similar to the rumen environment (S. Gilmore, Native Microbials Inc., San Diego, CA; unpublished data). Identifying novel, relevant microbial strains to create next-generation DFMs is important to aid in efforts to increase livestock production efficiency and support the growing human population. Additionally, DFMs may reduce reliance on feed additives that could promote antimicrobial resistance (National Research Council, 1980; Verraes et al., 2013). Therefore, the study objective was to evaluate the effectiveness of a new MFS comprising native rumen microorganisms on the performance parameters of mid-lactation dairy cattle.

All procedures were approved by the Iowa State University Institutional Animal Care and Use Committee (9-17-8601-B), and the study was conducted from May to August 2018. Forty-six lactating Holstein cows (mean ± SD: 629 ± 63 kg of BW; parity 1.63 ± 0.49; 119 ± 38 DIM; 45.11 ± 3.81 and 52.73 ± 4.85 kg/d milk yield for primiparous and multiparous, respectively) were housed in a single pen within a freestall barn at the Iowa State University Dairy Farm. All animals were given 4 ± 2 d to acclimate, during which they were trained to their individually assigned Calan gates (Calan Broadbent feeding system, American Calan). Cows were fed ad libitum once daily at 0645 h with a diet formulated to meet or exceed the predicted requirements (National Research Council, 2001) of energy, protein, minerals, and vitamins. Feed samples were collected weekly, condensed into a representative sample, and analyzed by Dairyland Laboratories Inc. (Arcadia, WI). The diet consisted primarily of corn silage (40.4% of DM), alfalfa hay (15.4% of DM), and ground corn (15.4% of DM), and the chemical composition on a DM basis was 26.9% starch, 16.8% CP, 29.6% NDF, 20.5% ADF; NE_L_ was 1.64 Mcal/kg of DM. Orts were collected daily at 0600 h using a Schuler wagon (model 125BF, Schuler Mfg. & Equip. Co. Inc.), recorded using a feed scale, and discarded. Cows were milked 3 times daily (~0500, 1400, and 2100 h), and yield was recorded using the Boumatic Smart parlor system and reported by PCDart dairy management software (Dairy Records Management Systems). Milk samples for composition analysis were obtained once weekly from all 3 milkings (Boumatic SmartControl metering system) and were stored at 4°C with a preservative (bronopol tablet; D&F Control Systems) until analysis by Dairy Lab Services (Dubuque, IA) using AOAC-approved infrared analysis equipment and procedures (AOAC International, 1995). Body weights and body condition scores were obtained once weekly after the 0500 h milking; BCS was determined by the same individual using the 5-point scale BCS Flowchart (Elanco Animal Health, 2009).

The trial consisted of 3 experimental periods (**P**); P1, which lasted 7 d, served as the baseline and yielded data for covariate analysis. During P2, which lasted 60 d, animals were assigned to 1 of 2 dietary treatment groups in a randomized complete block design to balance for milk yield, parity, and DIM: (1) control (**CON**; base TMR; n = 23; 8 primiparous and 15 multiparous) or (2) MFS (base TMR + 5 g/d of Galaxis; Ascus Biosciences Inc.; n = 23; 9 primiparous and 14 multiparous). Sample size was determined based on previous unpublished results involving this MFS; a 3-kg difference between groups with an SD of 3.5 kg could be detected with sufficient power (0.81) using a total sample size of 46 cows. Treatment was top-dressed and hand-mixed into the top one-third of the TMR. Finally, P3 lasted 7 d and served as a washout period, during which only the base TMR was provided ad libitum and no treatment was given. All cows had unlimited access to water for the duration of the experiment.

All milk yield (**MY**), ECM, and DMI data were condensed to weekly means before analysis. The duration of P2 was 60 d, so the final week of P2 was labeled as 8 + 4 d. Daily weighted averages for milk protein and fat were calculated by multiplying milk protein or fat percentage from each respective milking by that milking's MY. Yield (kg) was then summed for fat and protein, respectively, for each day. Fat, protein, and milk yields were represented in the ECM equation as described by VanBaale et al. (2005), using the following equation: ECM = [(0.327 × MY) + (12.95 × fat yield) + (7.2 × protein yield)]. Feed efficiency (**FE**) was determined as ECM/DMI.

Before analysis, response variables were checked for violations of normality and equal variance via inspection of histograms, quantile-quantile plots performed on initial modeling results, and Levene's test for unequal variance (Levene, 1960). Response variables were fitted to linear mixed effects models, in which the animal ID was specified as the subject, and an autoregressive covariance structure was imposed via the R (ver. 3.5; https://www.r-project.org/) package “nlme” (Pinheiro et al., 2020). Separate variance structures for each treatment group were imposed during modeling to account for violations of homoscedasticity; that is, violation of equal variance between groups. Response variables were analyzed using the following model:

$$Y_{ijklm}=T_{i}+W_{j}+L_{k}+P_{l}+T_{i}\times W_{j}+C_{m}+e_{ijklm},$$

where *Y_ijklm_* = response variable, *T_i_* = fixed effect of treatment, *W_j_* = fixed effect of time (week), *L_k_* = fixed effect of parity, *P_l_* = fixed effect of the average P1 production level per cow, *T_i_* × *W_j_* = interaction between treatment and time, *C_m_* = random effect of cow, and *e_ijklm_* = residual. From the resulting model, estimated marginal means (emmeans) were computed using Satterthwaite degrees of freedom to compare the difference in response between treatment and control across weeks of trial via the R package “emmeans.” Data are reported as emmeans and considered significant if *P* ≤ 0.05 and a tendency if 0.05 < *P* ≤ 0.10. Upon inspection of histograms and quantile-quantile plots, SCC were found to be non-normal and were log-transformed before analysis. The remaining response variables were confirmed to exhibit sufficient normality and thus were not transformed.

After the first round of modeling of the data set, an analysis of covariance (ANCOVA) was performed to model the relationship between post-administration (P3) milk response and treatment, with P1 production serving as a covariate. Following the discovery of a trending interaction effect between treatment and P1 production, the average P1 production values were used as cutoffs to create subsets of the data. At each cutoff, cows with a P1 production greater than this value were removed from the data set. Using these subsets, the percent change in MY and ECM between MFS cows and CON cows was calculated and then plotted for each cutoff (Figure 1). This analysis suggested that cows with a higher initial P1 production had a decreased response relative to that of cows with lower P1 production.

**Figure 1:**
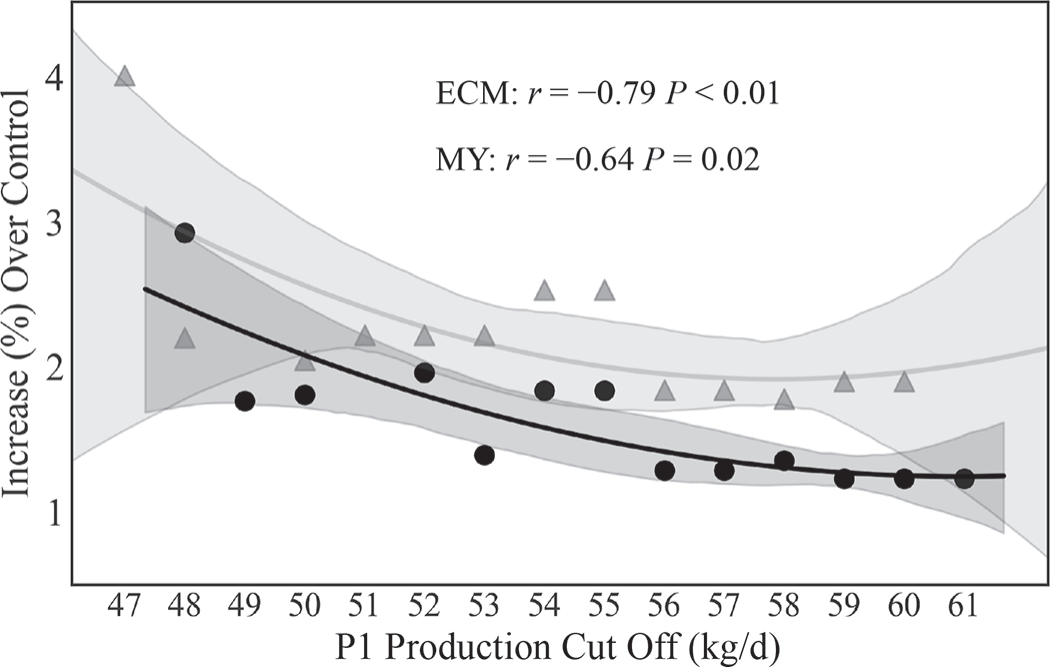
Regression showing the effect of covariate-based cutoffs on milk yield (MY; black circles) and ECM (gray triangles) response between treatment groups (n = 46). The percentage change in response over the control group (CON) was calculated using only those cows with an average covariate production level less than or equal to the production values along the x-axis. Shaded regions represent the dispersion of points around the associated regression line. Pearson correlation coefficients (r) and their associated *P*-values were also calculated and are shown. P1 = period 1 (before administration of microbial feed supplement).

Based on this analysis, cows were retrospectively partitioned into 2 groups based on their MY and ECM during P1, with cutoffs of 55 and 53 kg/d, respectively. Cows with a lower MY and ECM were categorized into milk production group (**MPG**) 1 (n = 34; 17 per treatment), and cows with a higher MY and ECM were categorized into MPG2 (n = 12; 6 per treatment). Groups were balanced for parity within treatment for MPG1 (with MFS and CON each having 8 primiparous and 9 multiparous) and considered sufficiently balanced in MPG2 (MFS, 1 primiparous and 5 multiparous; CON, 0 primiparous and 6 multiparous) given the available subjects. After partitioning, the analysis was rerun using the same method detailed above.

We detected no treatment differences associated with performance parameters such as ECM, MY, DMI, or milk composition in cows that were supplemented with MFS (*P* ≥ 0.23; Table 1). There were no treatment differences in milk protein yield, milk fat yield, or other metrics of milk composition (*P* > 0.15; Table 1). There was, however, an interaction between treatment and time on FE as treatment progressed (*P* = 0.03; Table 1). Overall, BW and BCS were similar between treatments (*P* ≥ 0.18; Table 1).

**Table 1:** Effect of microbial feed supplement (MFS) on performance variables during administration of MFS

Variable	Treatment^1^				*P*-value	
	CON	SD	MFS	SD	Treatment	Treatment × time^2^
All cows^3^						
Milk yield (kg/d)	45.95	1.50	46.09	1.62	0.96	0.45
DMI (kg/d)	24.67	0.80	24.99	0.73	0.73	0.23
Milk composition						
Fat (%)	3.27	0.10	3.34	0.17	0.61	0.15
Fat (kg/d)	1.51	0.05	1.54	0.07	0.68	0.32
Protein (%)	2.95	0.09	3.00	0.08	0.30	0.68
Protein (kg/d)	1.36	0.03	1.38	0.03	0.51	0.57
Lactose (%)	4.76	0.03	4.79	0.02	0.34	0.19
Other milk solids (%)	5.66	0.03	5.69	0.02	0.30	0.21
Total milk solids (%)	11.88	0.12	12.04	0.18	0.36	0.23
SCC (log)	3.86	0.15	4.08	0.32	0.32	0.67
MUN (mg/dL)	13.14	1.42	12.77	1.20	0.39	0.66
ECM (kg/d)	44.27	1.10	44.98	1.30	0.66	0.46
Feed efficiency (ECM/DMI)	1.80	0.05	1.81	0.04	0.93	0.03
BW (kg)	623	8	636	7	0.40	0.47
BCS	2.91	0.06	3.05	0.03	0.18	0.28
Milk production group 1 cows^4^						
Milk yield (kg/d)	43.68	1.52	44.75	1.44	0.10	0.35
DMI (kg/d)	24.73	0.82	24.82	0.76	0.30	0.42
Milk composition						
Fat (%)	3.27	0.15	3.40	0.17	0.35	0.21
Fat (kg/d)	1.43	0.05	1.53	0.07	0.10	0.34
Protein (%)	3.01	0.09	3.01	0.07	0.94	0.47
Protein (kg/d)	1.32	0.03	1.35	0.03	0.33	0.72
Lactose (%)	4.77	0.03	4.80	0.03	0.49	0.10
Other milk solids (%)	5.68	0.02	5.71	0.03	0.51	0.19
Total milk solids (%)	11.97	0.14	12.12	0.18	0.39	0.29
SCC (log)	3.66	0.19	3.86	0.35	0.43	0.74
MUN (mg/dL)	13.03	1.37	12.73	1.24	0.50	0.73
ECM (kg/d)	42.26	1.06	44.11	1.07	0.04	0.71
Feed efficiency (ECM/DMI)	1.75	0.05	1.79	0.15	0.24	0.25
BW (kg)	621	8	628	7	0.64	0.55
BCS	3.00	0.05	3.11	0.05	0.45	0.18
Milk production group 2 cows^5^						
Milk yield (kg/d)	52.54	1.53	50.48	2.35	0.10	0.60
DMI (kg/d)	25.78	0.87	25.66	1.01	0.60	0.12
Milk composition						
Fat (%)	3.23	0.09	3.19	0.16	0.59	0.95
Fat (kg/d)	1.72	0.06	1.61	0.11	0.22	0.94
Protein (%)	2.76	0.08	2.98	0.10	0.07	0.58
Protein (kg/d)	1.46	0.04	1.49	0.05	0.99	0.68
Lactose (%)	4.71	0.04	4.76	0.02	0.54	0.53
Other milk solids (%)	5.60	0.04	5.67	0.02	0.44	0.48
Total milk solids (%)	11.60	0.10	11.84	0.18	0.72	0.92
SCC (log)	4.64	0.26	4.59	0.24	0.65	0.67
MUN (mg/dL)	13.11	1.63	13.16	1.10	0.43	0.26
ECM (kg/d)	50.01	1.30	47.97	2.01	0.21	0.75
Feed efficiency (ECM/DMI)	1.96	0.07	1.88	0.05	0.19	0.28
BW (kg)	629	9	666	7	0.31	0.55
BCS	2.61	0.09	2.89	0.13	0.12	0.08

1 CON = control; MFS = 5 g/d Galaxis (Ascus Biosciences Inc.) administered.

2 Weeks in trial.

3 All cows on trial; CON (n = 23) and MFS (n = 23).

4 Milk production group 1 cows includes cows with an average baseline production level of 53 kg/d or less of ECM; CON (n = 17) and MFS (n = 17).

5 Milk production group 2 cows includes cows with an average baseline production level of 53 kg/d or more of ECM; CON (n = 6) and MFS (n = 6).

The model generated for MY displayed high variance for the random subject effect, indicating the presence of high intra-cow variation in response to treatment. Additionally, the fixed effect of average P1 production (*P_l_*) was strongly significant across our mixed model results (*P* < 0.01). An analysis of covariance was then performed, fitting post-administration (P3) milk production against treatment using P1 milk production as a covariate. We observed a trending effect for treatment (*P* = 0.07) and an interaction between treatment and P1 production (*P* = 0.07), suggesting that initial production might have an effect on the efficacy of the MFS. To explore this relationship further, a second-order polynomial regression was fit to covariate subsets of the data and the observed increase in milk production (Figure 1). Energy-corrected milk during P1 correlated with a percent change, with a Pearson correlation coefficient r = −0.79 and *P* < 0.01. Similarly, MY during P1 correlated with a percent change, with r = −0.64 and *P* = 0.02. We investigated this effect temporally using 3-dimensional surface plots (Figure 2). The ECM plot (Figure 2A) shows that lower-producing animals had a more robust response over time than higher-producing cows. The MY plot (Figure 2B) shows a similar trend to ECM. The DMI plot (Figure 2C) shows that higher-producing cows had a larger decrease in DMI over time compared with lower producers.

**Figure 2:**
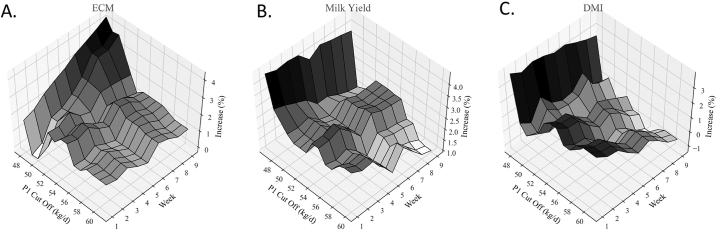
Percent increase over control group of treatment group receiving the microbial feed supplement (MFS) represented as a function of time (Week) and ECM production during period 1 (P1; before administration of MFS) for (A) ECM, (B) milk yield, and (C) DMI during period 2 (when MFS was administered; n = 46). The initially lower-producing animals had a greater percentage improvement over time than those that had initially higher production.

Because the effects of MFS appeared to be influenced by P1 production values, we grouped cows into 2 separate data sets. Cows producing <53 kg/d of ECM in P1 were categorized into MPG1 (n = 34; 17 per treatment), and cows producing ≥53 kg/d of ECM were categorized into MPG2 (n = 12; 6 per treatment). This value was chosen because it provided balanced groups for both MPG1 and MPG2, and also served as an inflection point in the polynomial regression involving ECM (Figure 1), where subsets beyond this cutoff exhibited diminishing returns in percent change. Groups were balanced for parity and the variance in DIM between the groups, being 116 ± 35 for MPG1 and 129 ± 48 for MPG2.

Cows in the MPG1 receiving the MFS had 1.85 kg/d increased ECM relative to CON cows (*P* = 0.04; Table 1). Similarly, milk and milk fat yield from cows receiving MFS trended to be 1.07 kg/d (*P* = 0.10; Table 1) and 0.10 kg/d (*P* = 0.10; Table 1) higher compared with that of CON cows, respectively. Milk lactose exhibited a trending treatment by time interaction (*P* = 0.10; Table 1). Supplementation with MFS had no detectable differences on milk protein, milk solids, SCC, MUN, DMI, FE, BW, or BCS (*P* ≥ 0.18; Table 1).

Milk yield for MPG2 cows tended to be decreased by 2.06 kg/d in MFS cows relative to CON (*P* = 0.10; Table 1). Milk protein percentage exhibited a trending increase of 0.22 percentage points (*P* = 0.07; Table 1). Additionally, there was a trend for a treatment by time interaction for BCS (*P* = 0.08; Table 1), a scenario suggesting that increased ME was used for non-mammary purposes, although overall BW was unaffected (*P* > 0.31; Table 1) by treatment. There were no overall treatment differences associated with MFS for the remaining production parameters in MPG2 cows (*P* ≥ 0.12; Table 1).

The influence of MFS on production characteristics appears to be linked to the level of milk production, because supplementation had different effects on performance parameters of higher- and lower-producing cows. We showed that MFS seemed to have a beneficial effect on milk yield and milk components in MPG1 cows and tended to decrease milk yield in MPG2 cows. Further exploration of the relationship between this MFS and production level is merited. One reason may be that milk synthesis in the MPG2 was not limited by energy and component precursors, and any additional energy provided by the MFS was not directed toward increasing milk production, as it may have been for MPG1 cows. As previously stated, in the absence of improvements to milk production, the effects of MFS appear to manifest elsewhere, as we observed in the trending for increased BCS in MPG2 cows. Furthermore, similar to previous articles investigating milk production, different responses might depend on the particular energetic needs of the cow (Bradford and Allen, 2004). These energetic requirements affect many factors of the cow's physiological processes and are intrinsically tied to lactation, suggesting that understanding these effects may play an important role in the application of MFS (Ferrell and Jenkins, 1985; Freetly et al., 2006).

We conducted a washout period to determine whether there was a residual effect of MFS. We observed no differences in production parameters during the 1-wk washout period with respect to the previous weeks of administration. This is consistent with previous independent studies that show that the microbiome takes longer than 1 wk to revert to an original state (Weimer et al., 2010).

When all cows in the trial were considered as a whole, MFS did not affect MY, ECM, DMI, BW, or BCS. Administration of MFS did, however, tend to increase FE compared with CON cows. When the animals were separated pre-trial into higher- and lower-producing groups, a treatment effect was revealed. Cows in the lower-producing group fed MFS had increased ECM and showed a tendency for increased milk and fat yields and lactose over time. Supplementation with MFS tended to decrease MY, increase protein concentration, and increase BCS over time in cows in the higher-producing group.
